# Preventive effect of probiotics on periodontal ligament in a rat model of anorexia nervosa

**DOI:** 10.1038/s41598-025-02610-x

**Published:** 2025-06-02

**Authors:** Marta Rizk, S. Häck, S. Brenji, I. Knaup, C. Niederau, F. Kiessling, N. Marx, J. Moellmann, F. Kahles, T. Pufe, C. Apel, J. Kern, J. Seitz, L. Käver, C. Voelz, M. Wolf, S. Trinh, R. B. Craveiro

**Affiliations:** 1 Department of Orthodontics, Uniklinik RWTH Aachen, Aachen, Germany; 2 Institute for Experimental Molecular Imaging, Uniklinik RWTH Aachen, Aachen, Germany; 3 Department of Internal Medicine I - Cardiology, Angiology and Intensive Care Medicine, Uniklinik RWTH Aachen, Aachen, Germany; 4 Department of Anatomy and Cell Biology, Uniklinik RWTH Aachen, Aachen, Germany; 5 Department of Biohybrid and Medical Textiles, AME-Institute of Applied Medical Engineering, Uniklinik RWTH Aachen, Aachen, Germany; 6 Department of Prosthodontics and Biomaterials, Center for Implantology, Medical Faculty, RWTH Aachen University, Aachen, Germany; 7 Department of Child and Adolescent Psychiatry, Psychosomatics and Psychotherapy, LVR-University Hospital, University of Duisburg-Essen, Essen, Germany; 8 Institute of Neuroanatomy, Uniklinik RWTH Aachen, Aachen, Germany; 9 Institute of Functional and Applied Anatomy, Hannover Medical School, Hanover, Germany

**Keywords:** Activity-based anorexia model, Anorexia nervosa, Probiotics, Periodontal and alveolar bone remodeling, Periodontal ligament, Tomography, Dentistry, Periodontology, Bone, Experimental models of disease, Preclinical research, Dentistry

## Abstract

The rising prevalence of anorexia nervosa (AN), especially among adolescents, and the limited understanding of its effects on the periodontium often hinder decision making in dentistry and periodontology. As an adjunct to periodontal therapy, probiotic administration has shown promising effects on oral health by decreasing pathogen counts and altering the immune response. This study thus investigates changes in morphology and remodelling of alveolar bone and periodontal ligament (PDL) due to an AN-like condition, along with potential protective effects of probiotics, using a rat model. Three-week-old female Wistar rats were divided into three groups: a control group with ad libitum food and two groups undergoing an activity-based anorexia (ABA) model. One ABA group received oral multi-strain probiotics during starvation. After five weeks, all rats were sacrificed for ex-vivo micro-CT scans of the maxilla and mandible to assess alveolar bone and PDL morphology, as well as histological evaluations for PDL fibre vitality and structural organization. The data were statistically evaluated by one-way ANOVA with Tukey’s post-hoc test or by Kruskal–Wallis with Dunn’s test for parametric or nonparametric data, respectively. The results showed no structural changes in alveolar bone caused by either ABA or probiotic treatment; however, the ABA group exhibited a significant reduction in PDL thickness, which could not be reversed by probiotic treatment. Despite this, histological analysis indicated improved connectivity and density of PDL fibres in the probiotic group compared with the ABA-only group. No differences were found between the mandible and maxilla. In conclusion, while probiotics did not prevent PDL thinning, they enhanced its composition/vitality compared to the ABA condition alone.

## Introduction

In today’s beauty-conscious society, anorexia nervosa (AN) is one of the leading eating disorders, as evidenced by its ever-increasing prevalence, especially among adolescents^1–3^. As this group represents a substantial proportion of orthodontic patients, it is important to investigate the effect of eating disorders on the development of the periodontium in adolescents^4,5^.

This condition of severe underweight is associated with, among other things, low bone mass, impaired bone structure and reduced bone strength, all of which contribute to an increased risk of fracture. Adolescents with AN have reduced rates of bone mineral accrual when compared to healthy individuals^6^. Additional consequences of pathological weight loss are often vitamin deficiencies, especially vitamins essential for bone mineralization and regeneration, such as vitamins C and D^6,7^. Other vitamin deficiencies may affect the development and vitality of the neural and cardiovascular systems, soft tissues and other major organs^8,9^. AN has been shown to lead to an increased bone loss due to increased levels of the peptide hormone PYY, which acts as a negative regulator of osteoblastic bone growth^6,10^. Several studies have shown morphological and compositional changes in the bones of patients with AN, such as decreased bone density, trabecular thickness, and increased porosity^11,12^. These parameters have mostly been studied in the tibia or lumbar spine. However, it is unclear whether similar changes occur in the periodontium and whether soft tissues such as the periodontal ligament (PDL) are also affected^7^.

The knowledge of the impact of AN on the bone metabolism raises the question of how much this condition affects the bone and soft tissue remodelling of the periodontium in AN patients, especially during orthodontic treatment and dental interventions. A deeper understanding of the inter-correlation between this eating disorder and bone homeostasis or changes in the PDL as a stress absorber is a key factor for more effective decision making in dental clinics. In addition, oral comorbidities of eating disorders, such as dental erosion, caries and changes in salivary composition often observed in patients with AN, play an important role in predicting outcome before and during orthodontic treatment^7,13^.

Recent animal models using high-resolution micro-computer tomography (micro-CT), and other techniques provide a reasonable opportunity for such reproducible investigations. Although AN is considered a mental disorder and cannot be fully mimicked in animals, its physical manifestation has been well established using a combination of controlled weight loss and exercise in a so-called activity-based anorexia (ABA) model^14,15^.

Furthermore, the positive effects of probiotics as a preventive measure against bone degradation or mineral loss, as demonstrated in numerous previous studies, suggest the potential for probiotic administration as an adjunct therapy to orthodontic and other dental procedures. This could help to reduce treatment-induced bone and mineral loss and other degradations of the periodontium. Several previous studies have demonstrated a benefit of such supplementation through a suppression of bone loss^16–21^; an improved absorption of calcium and phosphate^22,23^; an increase in bone mineral density or alveolar bone volume^24,25^. However, there is still a lack of clarity regarding the morphological changes in the periodontium of AN patients with or without probiotic supplementation. This is due to the predominant focus of such studies on the vertebrae or femur/tibia. Regarding inflammatory conditions, several studies have confirmed a positive effect of probiotic treatment on gingival inflammation, bleeding on probing, and reduction in oral pathogen counts^26–29^. The potential beneficial effect of probiotics against bacteria has been shown through alterations in the oral microbiome and modulation of host responses to inflammation^30^. Probiotic use as adjuvant therapy to periodontal treatment has been shown to enhance reduction of periodontopathogenic species and inflammatory responses in periodontal tissue^28,31,32^ Yet, to our knowledge, no study has investigated the influence of an AN-like condition combined with preventive probiotic therapy on the periodontal ligament.

The objective of the current study is to investigate the potential impact of AN-like conditions on alveolar bone and periodontal ligament tissue using the ABA rat model. Moreover, we aim to investigate the impact of probiotic administration in minimizing those possible impacts. This will be achieved by micro-CT imaging and histological evaluation. This study aims to lay the groundwork for the development of a model of AN that will provide crucial insights into the associated risks and potential interventions during dental treatment.

## Methods

### Rat model

Eighteen female Wistar rats (3 weeks old) were purchased from Janvier Labs (RjHan:WI; Hannover, Germany) and arrived at the animal facility with a body weight of 104.3 ± 7.2 g. All animals were housed individually in type IV polysulfone cages (Tecniplast GmbH, Hohenspeißenberg, Germany) under controlled room temperature (± 23 °C) and humidity (± 55%) with a 12-h light/dark cycle. The animal facility was specific pathogen free according to DIN ISO 9001:2008. All procedures were approved by the Governmental Animal Care and Use Committee (Recklinghausen, Germany; approval number 81-02.04.2021.A183) and were performed in accordance with German legislation on animal studies following the Guide for the Care and Use of Laboratory Animals (NIH publication, 8th edition, 2011) and the 2010/63/EU Directive on the protection of animals used for scientific purposes (Official Journal of the European Union, 2010). The animals used were part of a larger study conducted at the Institute for Neuroanatomy, University Hospital, RWTH University Aachen, Germany. Sample size calculation was based on a two-tailed Satterthwaite t-test for unequal variances, assuming a low dropout rate (maximum 5%). This study is reported in accordance with ARRIVE guidelines.

### Study design

**Fig. 1:**
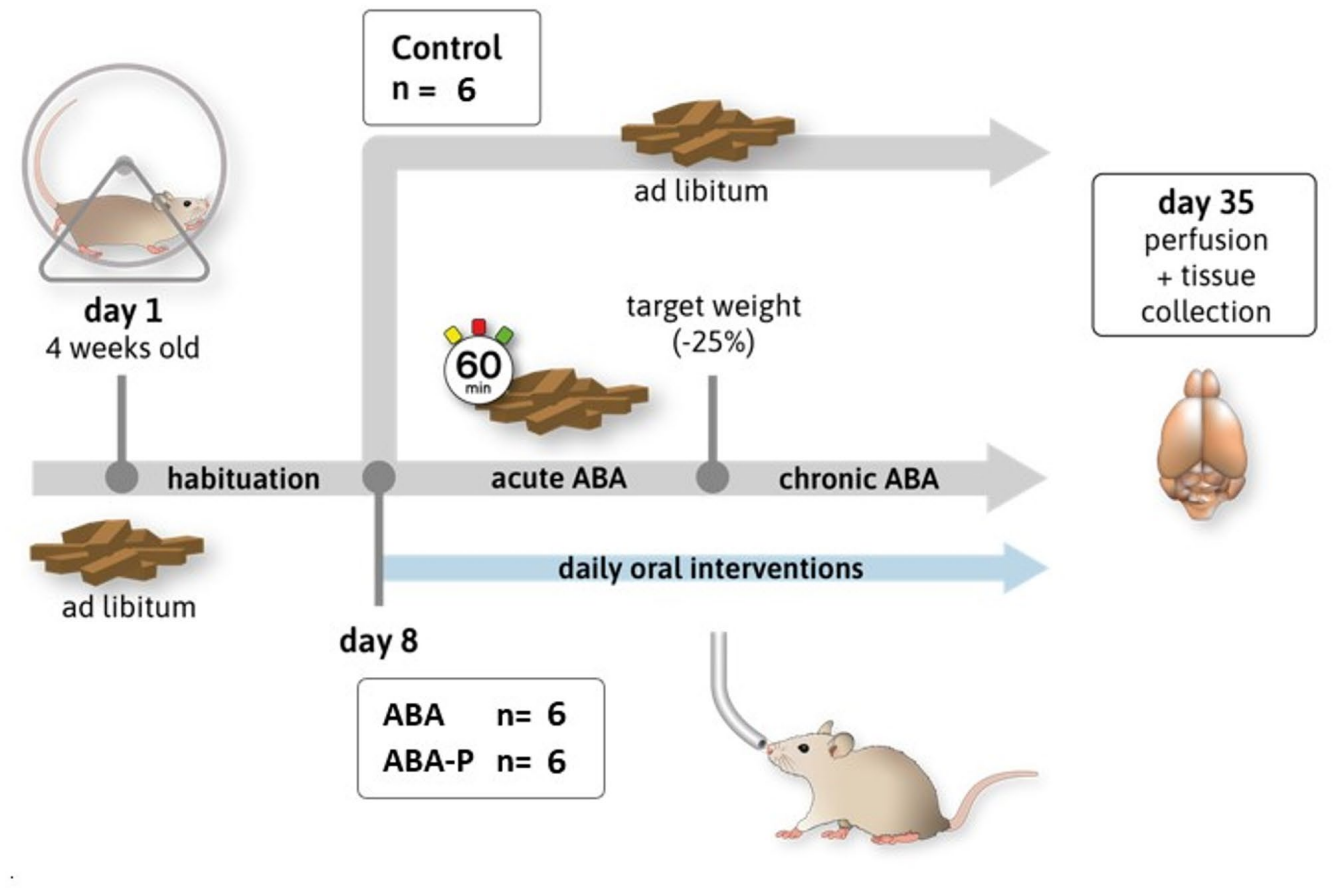
Graphical representation of the animal experiment. After 8 days of habituation with ad libitum access to food, the ABA and ABA-P groups underwent the acute and later chronic ABA phase with controlled feeding and daily activity. Both groups received daily oral interventions with water (ABA) or probiotics (ABA-P).

Rats were randomly divided into three groups (Fig. 1): control (C), intervention with vehicle – water intervention (ABA)^14,15^ and probiotic intervention (ABA + P)^33^. After a one-week acclimation period for all animals, the ABA and ABA + P groups were subjected to the activity-based anorexia (ABA) model with food access restricted to 1 h per day (1–2 pm), while the C group had ad libitum access to food throughout the experiment. All rats had access to an in-cage treadmill throughout the experiment. After reaching the target body weight loss of 25% for ABA and ABA + P groups, chronic starvation was initiated. During this phase, the amount of food was individually adjusted for each rat to ensure a stable body weight. During the acute and chronic starvation periods, the ABA + P group received a daily oral administration of a multi-strain probiotic from Allergosan (Graz, Austria; OMNi-BiOTiC Stress Repair: *Lactobacillus casei W56, Lactobacillus acidophilus W22, Lactobacillus paracasei W20, Bifido-bacterium lactis W51, Lactobacillus salivarius W24, Lactococcus lactis W19, Bifidobacterium lactis W52, Lactobacillus plantarum W62 and Bifidobacterium bifidum W23*, dosed as 1 g dissolved in 1 ml water). The ABA group received 1 ml of water orally daily as a control intervention. At the end of the protocol (day 35), all groups were euthanised with 100% isoflurane (Piramal, Mumbai, India) and transcardially perfused.

### Micro-CT analyses

The right sides of both jaw bones, maxilla and mandible, were scanned by a Skyscan 1272 μCT scanner (Bruker MicroCT, Kontich, Belgium) at 60 kV, 166 μA, with a rotation step of 0.1°. A nominal resolution of 6^3^ cubic micrometers was chosen. The data were then reconstructed using NRecon software (Bruker MicroCT, Kontich, Belgium). The data sets were 3Dregistered to a reference to ensure the reproducible position of the studied volume using DataViewer software (Bruker MicroCT, Kontich, Belgium), as described in detail in the previous work^34^.

**Fig. 2:**
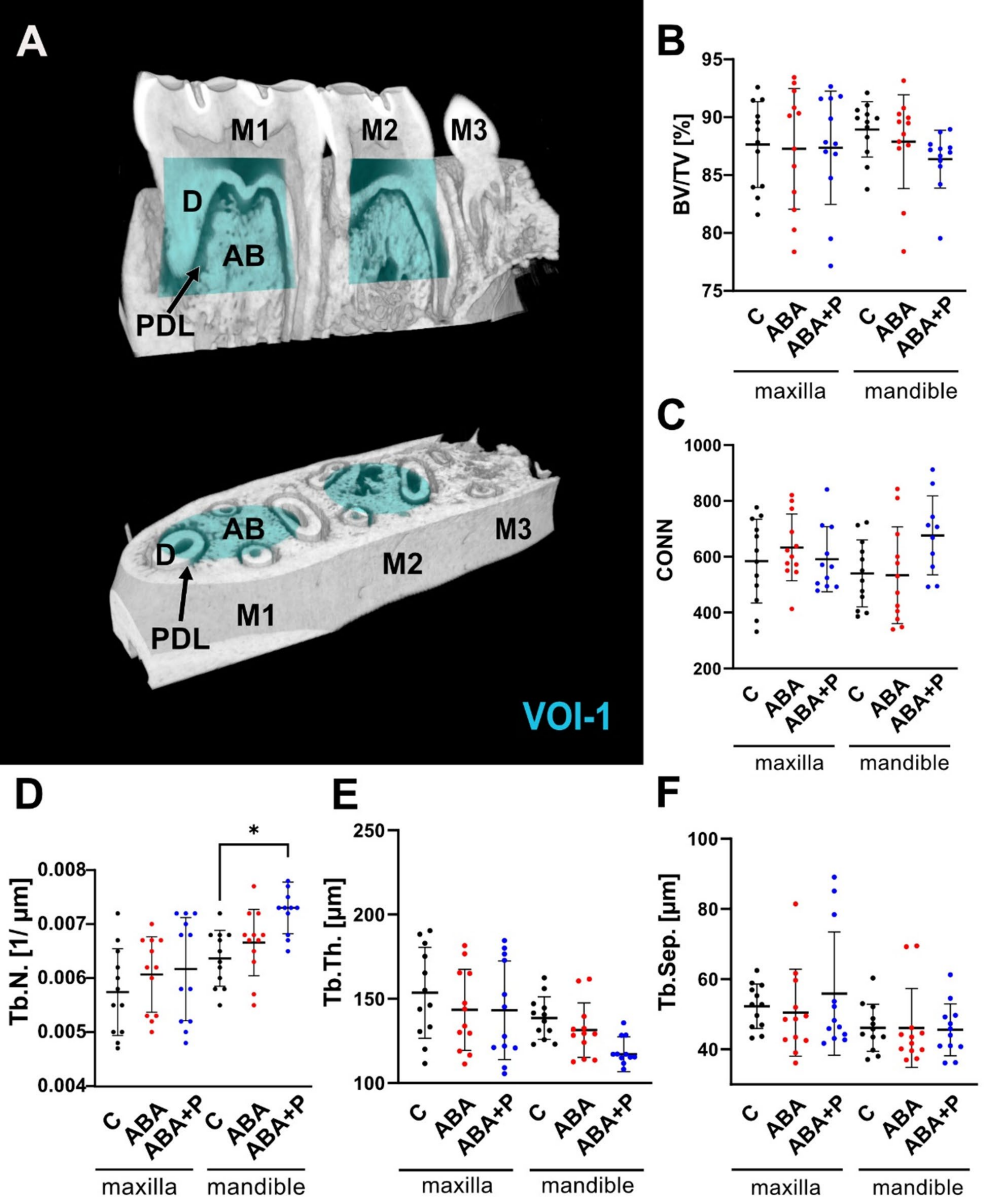
Evaluation of the alveolar bone morphology in the M1 and M2 sockets of the maxilla and mandible: (**A**) morphological parameters were estimated from the cylindrical volume of interest (VOI-1) of M1 and M2. Statistical evaluation of the bone parameters revealed relatively stable alveolar bone morphology under ABA condition independent of probiotic therapy: (**B**) BV/TV—bone volume/total volume, (**C**) CONN—trabecular connectivity, (**D**) Tb.N.—trabecular number, (**E**) Tb.Th.—trabecular thickness and (**F**) Tb.Sep.—trabecular separation, for all studied groups: C (control), activity-based anorexia model (ABA) and ABA + P (ABA + probiotics). M1—1st molar, M2—2nd molar, M3—3rd molar, D—dentin, AB—alveolar bone, PDL—periodontal ligament; *p < 0.05.

For the morphological study of the alveolar bone changes, two cylindrical volumes of interest (VOI-1)^34,35^, including the alveolar bone from the furcation up to 1 mm in apical direction, were placed in the first and the second molar tooth sockets (M1 and M2, respectively) (Fig. 2). A larger cylindrical VOI (VOI-2), which included the entire PDL space of M1, was used to study the PDL. All analyses were performed in CTan (Bruker MicroCT, Kontich, Belgium).

### Morphology of alveolar bone

Bone parameters were estimated using a combination of algorithms available in the software (CTan) and described elsewhere^34^. After a constant-based thresholding, a seed method was used for the bone segmentation within a VOI. To define the total volume, a closing algorithm was used to remove open and closed pores. To focus only on the fine bone microstructure, large porous structures entering the VOI from the apical side, defined by a primitive ROI at the bottom of the VOI, were removed from the analysis. Several bone morphological parameters were then estimated.

### PDL thickness and volume

Applying the seed algorithm, the tooth was virtually removed from the VOI-2. The outer boundaries of the PDL were then defined after the closing of the open pores of the alveolar bone using the closing method. The inner boundaries were defined as the boundaries of the tooth (root). The PDL was thus defined as the space within the binarized VOI-2 between the roots and the open-pore free alveolar bone. The algorithm for trabecular analysis was then applied to estimate the so-called PDL thickness.

### Histology

The left sides of the mandibles were collected and fixed in 3.7% paraformaldehyde solution for at least 24 h. The samples were decalcified with 10% Tris buffered ethylenediaminetetraacetic acid (EDTA, pH 7.4; Morphisto, Offenbach, Germany) on a shaker at room temperature for 8 weeks. The buffer was renewed every 2 days. After decalcification, each sample was embedded in paraffin and sectioned for histological analysis.

### Hematoxylin and eosin staining

To verify the findings of µCT analysis, serial transversal sections with of a thickness of 2.5 µm thickness were stained with hematoxylin and eosin (Epredia™ Signature Series™ Hematoxylin 7211, Thermo Fisher Scientific, Massachusetts, USA and Eosin G-Solution 3137.2, Roth, Karlsruhe, Germany, respectively).

### Collagen staining

Sharpey’s fibres from the PDL were stained with Sirius Red and Fast Green according to the manufacturer’s instructions (Sirius Red/Fast Green Collagen Staining Kit, 9046, Chondrex, Woodinville, USA). The sections were then coverslipped with Aquatex (1.08562.0050, Merck, Darmstadt, Germany). To quantify collagen staining, the first root was photographed and measured using Zen software (version 3.3 Zeiss, Jena, Germany) and the mean intensity value was analysed over an area of 20,000 µm^2^. Two molar sections were randomly selected from at least three animals for histological analysis.

### Statistical analysis

After conducting the Shapiro–Wilk normality test, the differences between all three groups in morphological parameters as well as in PDL thickness and volume estimated by micro-CT, and in collagen intensity were evaluated by one-way ANOVA with Tukey’s post-hoc test for normally distributed data or Kruskal–Wallis with Dunn’s multiple comparison test for data not following the normal distribution (α = 5%). Statistical analysis was performed using GraphPad Prism (GraphPad Software, Boston, USA, version 9.4.1). Results are presented as the means and standard deviations.

## Results

### Morphology of alveolar bone remains unchanged

Morphological examination of the alveolar bone in the M1 and M2 sockets (Fig. 2A) revealed no significant changes in the bone morphology under starvation conditions, irrespective of the additional probiotic intervention (Fig. 2B–F). Interestingly, in the mandible, a slight tendency of structural change towards alveolar bone with a higher number of thinner trabeculae can be seen under the probiotic supplementation (*p* = 0.0388). However, these changes are mostly within the experimental error limits.

### Thinning of the periodontal ligament by ABA

**Fig. 3:**
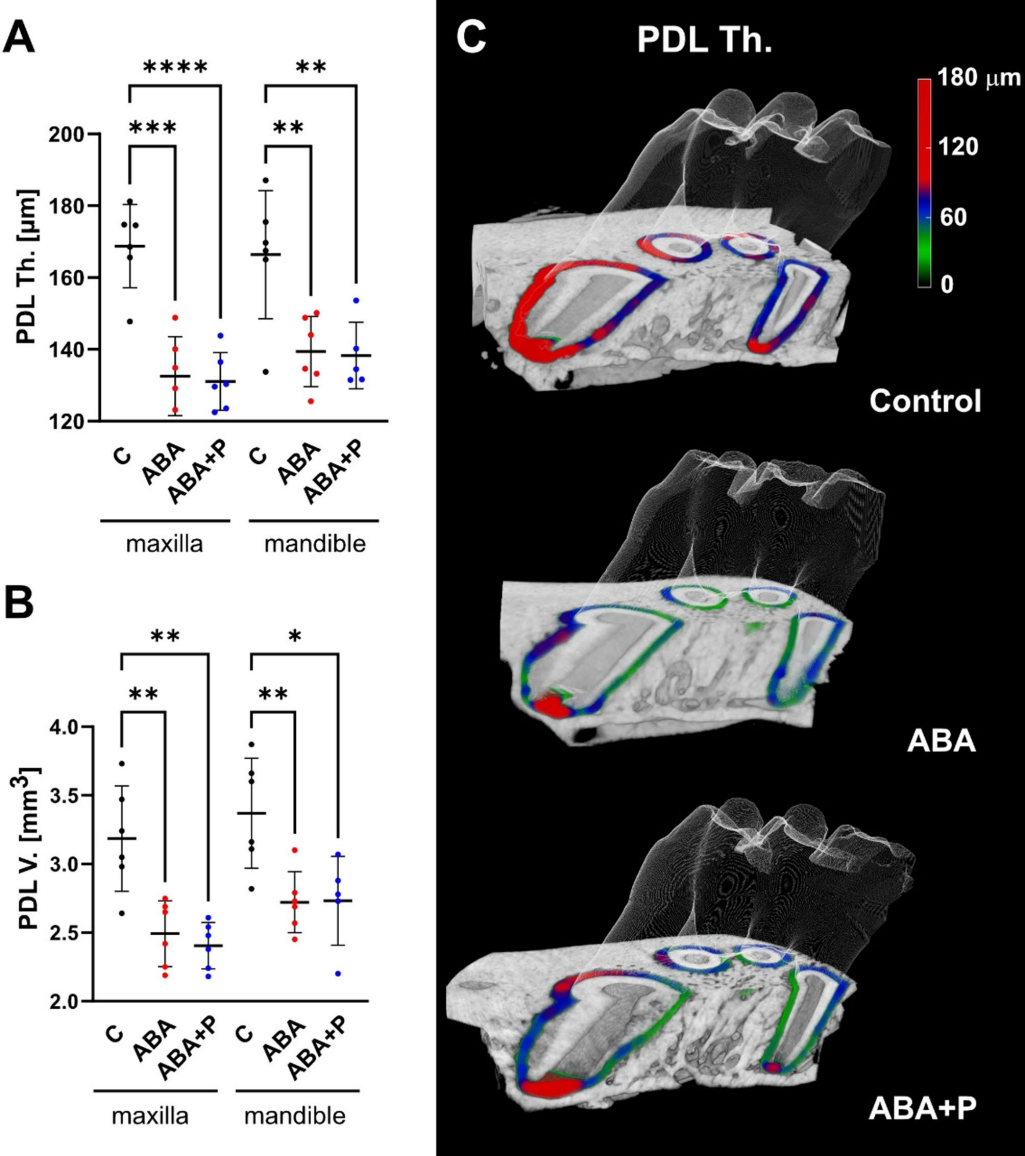
Analysis of PDL thickness and volume of M1 tooth in maxilla and mandible. A strong reduction of PDL thickness (**A**) and PDL volume (**B**) was found in the ABA group in both maxilla and mandible. This reduction could not be restored by probiotic administration. (**C**) 3D rendering of the VOI-2 with a color mapped PDL thickness shows the thinning of the PDL (predominantly blue/green areas) in the ABA and ABA + P groups compared to the control (prevalently red). *p < 0.05, **p < 0.01, ***p < 0.001, ****p < 0.0001.

According to the analysis in the full volume of the M1 socket, the PDL underwent a severe deterioration due to starvation conditions. Its thickness (PDL Th.) as well as its volume (PDL V.) were evidently reduced in ABA group in maxilla (PDL Th.: *p* = 0.0001; PDL V.: *p* = 0.0051) as well as in mandible (PDL Th.: *p* = 0.0052; PDL V.: *p* = 0.0099). This thinning was found to remain also in the probiotic group in maxilla (PDL Th.: *p* < 0.0001; PDL V.: *p* = 0.0014) and mandible (PDL Th.: *p* = 0.0056; PDL V.: *p* = 0.0175), thus no protective mechanism of probiotics against the PDL thinning could be detected by micro-CT (Fig. 3A,B). The 3D depictions of the PDL thickness in the colour-mapped image (Fig. 3C) show that the PDL thinning developed consistently in all its areas.

### Probiotic treatment shows a protective effect against loss of PDL fibre density

**Fig. 4:**
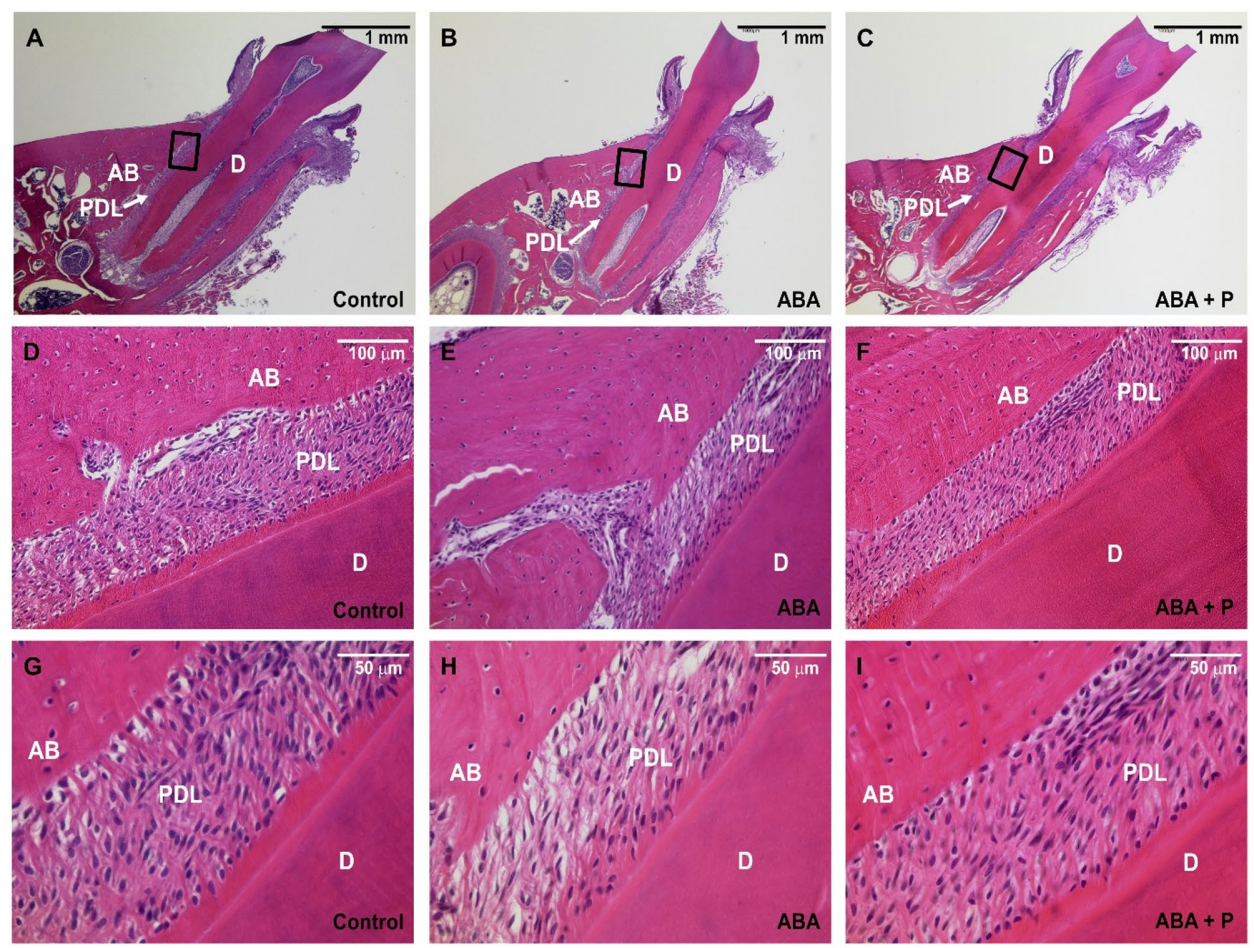
The PDL space around the 1st molar (M1) was evaluated histologically using HE staining and three magnifications. The PDL area was clearly reduced in both activity-based anorexia (ABA) (**B, E, H**) and ABA + P (ABA + probiotics) (**C, F, I**) groups compared to the control (**A, D, G**)**.** Histograms with the highest magnifications (scale 50 μm) revealed that the ABA group showed a strong loss of PDL fibres compared to the control group. In the ABA + P group, this structure was visibly repaired by the concomitant probiotic treatment. AB—alveolar bone; PDL—periodontal ligament; D—dentin.

Histological evaluations confirmed previous observations from 3D analysis by computer tomography (Fig. 4). A clearly thinner PDL space was observed in both the ABA and ABA + P groups compared to the control. Additionally, histology revealed a severe loss of PDL fibres in the ABA group, leading to less compact filaments with a disturbed orientation. This structure was visibly repaired by the additional probiotic treatment in the ABA + P group, although the ‘squeezing’ of the PDL space could not be avoided.

### Compactness of the PDL collagen reduced in ABA and restored in ABA + P group

**Fig. 5:**
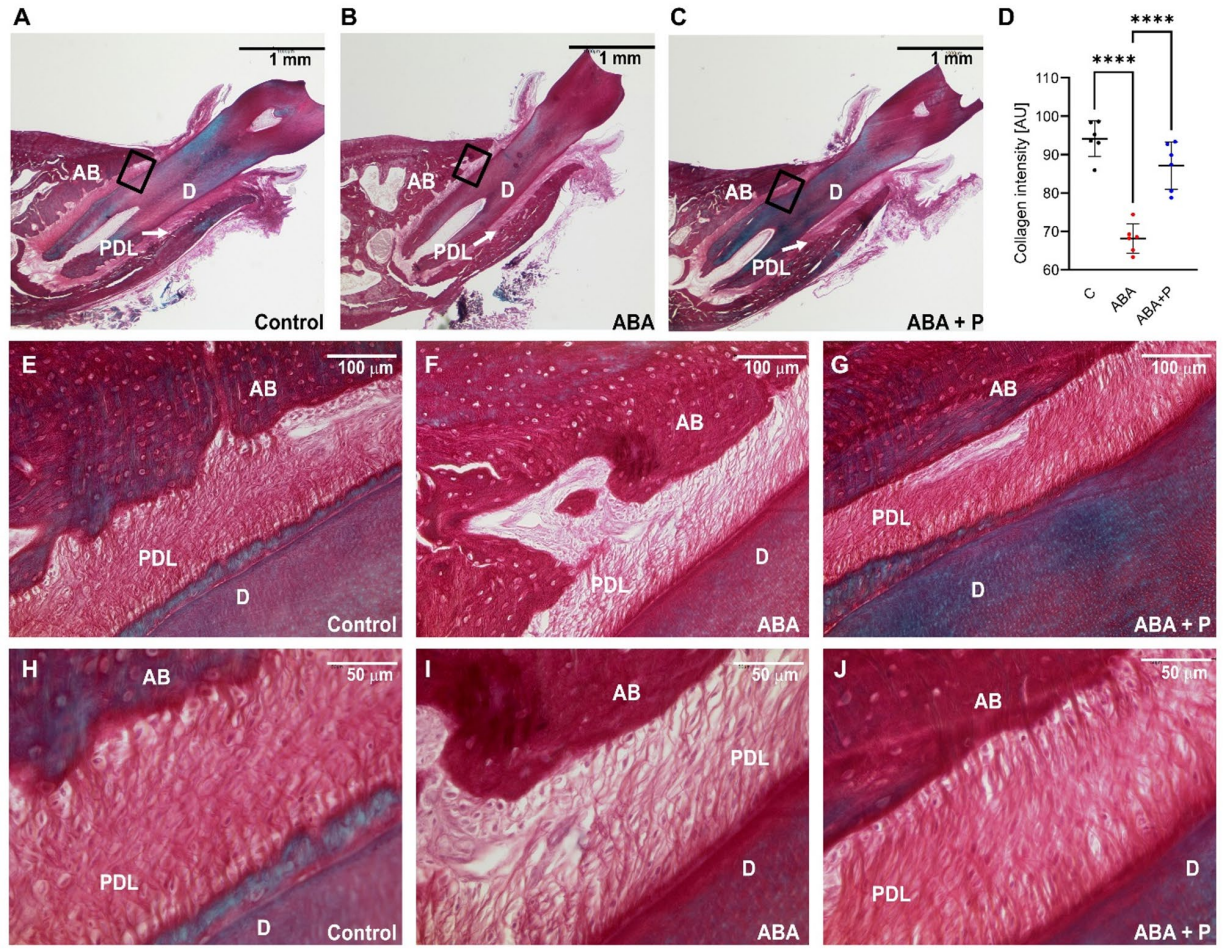
Collagen staining of the 1st molar space revealed less dense PDL fibres in the activity-based anorexia model (ABA) group while its structure remained unchanged under probiotic supplementation (ABA + P). (**A–C**) Overview of transverse sections of the periodontium in control, ABA and ABA + P groups, respectively. (**D**) Quantitative estimation of collagen intensity confirmed the preventive effect of probiotics against collagen degradation under ABA conditions. (**F, I**) At higher magnification, a strong loss of collagen fibres in the ABA group compared to the control (**E, H**) can be seen. In the ABA + P group (**G, J**) this structure was visibly repaired by the additional probiotic treatment. AB—alveolar bone; PDL—periodontal ligament; D—dentin. ****p < 0.0001.

Collagen staining was also performed, and similar observations observed by HE staining were confirmed. The collagenous structure of the PDL is apparently degraded by weight loss in the ABA group but showed relatively unchanged density and vitality when probiotic treatment was applied in the ABA + P group (Fig. 5).

## Discussion

This study provides the first observations on the changes in alveolar bone and periodontal ligament under anorexia nervosa-like conditions in rats and additional probiotic treatment. The data presented here provide a foundation for further research into the periodontal changes associated with AN and the potential preventive effects of probiotics in individuals with this condition.

Although several previous studies in patients with AN or rodents under dietary conditions^6,11,12,36^ have concluded an alteration in long bone morphology and density, no changes in the microstructure and porosity of alveolar bone were found in this study. The beneficial effect of probiotic supplementation on mineral bone density and bone volume fraction in the femur, tibia and spine of intact rodent models was also shown in a systematic study by Yumol et al.^37^. Such an observation suggests a possible difference in how caloric restriction affects bone homeostasis and metabolism in the long bone compared to the alveolar bone.

Since the ABA-unaffected morphology of the alveolar bone was maintained even after preventive supplementation with probiotics, we attribute this lack of effect of probiotics on alveolar bone morphology to the persistence of bone architecture after starvation periods. This finding is in line with the literature. As a few studies revealed no effect of probiotics in healthy population and no additional improvement over the control group with supplemental probiotic treatment^21,24,38,39^, the general view remains that there is no evidence of benefit from over supplementation in such individuals. Their protective role against bone loss in the femur, vertebrae or alveolar bone has only been confirmed in animal models with significantly impaired bone homeostasis due to ovariectomy, sex-steroid deficiency, periodontitis or other generalised disorders^17–19,21,24,38,40,41^. The preventive effect of probiotics against lumbar spine bone loss has also been demonstrated in postmenopausal women^20^. However, all these studies only confirm the preventive effect of probiotics against hormonal or inflammatory bone changes. Evidence for a beneficial influence of probiotics on bone homeostasis in healthy individuals is lacking. Although probiotics appear to be a promising tool for rebalancing the oral-gut microbiome as an armour against periodontal disease^42^ the advantage of such therapy lies in its preventive potential against degradation processes due to systemic diseases.

In addition to the alveolar bone, a healthy periodontal ligament is crucial for a resistant periodontium and its remodelling during clinical dental interventions in the alveolar bone. In a healthy periodontium, PDL consists of parallel oriented bundles of collagen fibres that act as a transport for stress in the periodontium during any external force application. However, it remains unclear how regular PDL fibre bundle arrays are formed and remodelled over long distances from the cementum to alveolar bone^43,44^.

To our best knowledge, there has been no study of the effect of AN on the PDL so far^7^. Our results showed the clear evidence of thinning of the PDL and weakening of the collagen network under a dietary condition, similar to AN. In addition to the geometry of the PDL, structural changes in the periodontal fibres can lead to different viscoelastic properties and affect the mechanical response^45,46^. It is therefore important to understand the factors that lead to any PDL structural changes, morphological remodelling or degradation of the PDL. This work therefore provides a new insight into the effect of this disease on the structure of the PDL, raising the question of whether similar effects menace in other eating disorders or under malnutrition or whether also other factors are also imperative in the PDL remodelling under dietary restrictions.

The degradation of PDL due to the ABA protocol found in this work raises the question whether, in addition to direct malnutrition, any vitamin deficiency may play a role in such morphological changes and degradation of the periodontium, as has been found previously^47–49,49^. Vitamin C has been shown to play a role as a regulator of collagen synthesis while enhancing the osteoblastic differentiation of PDL cells^50^. Vitamin D is known to play a role in bone resorption and helps to regulate the production of type I collagen^48,51^. In addition, possible hormonal changes caused by AN, such as oestrogen deficiency, or other dietary factors have been associated with reduced bone mineral density and an increased risk of osteoporosis in AN patients^52^. Though, further investigations are necessary to elucidate such possible underlying deficiencies.

The vitality of the PDL determines its mechanical properties^45^ and is crucial for proper stress absorption and redistribution, as well as in the prevention of tooth loss^53^. A finite element analysis study of a three-root tooth-PDL-bone complex showed that a small spatially varying geometric adjustments in the thickness of the PDL can cause a significant change in tooth reaction movement and in PDL/bone strain/stress distribution^46^. Coughi et al.^54^ found a correlation between the PDL thickness and a representative root dimension and thus also with the biological response to force application in Wistar rats. Mechanical strength of PDL fibres has been shown to be reduced by high carbohydrate diet while dependent on the collagen fibre components^45,55^. All these findings, together with our results, point to the importance of PDL dimension and vitality and the major influence of its changes on the mechanical properties of the periodontium.

Although our volumetric evaluations of the PDL showed that probiotic treatment could not prevent the thinning of this tissue, the clearly restored density of PDL fibres found by histology proves the potential of probiotics an adjunctive therapy in conditions where PDL degradation may occur. It is questionable whether the prebiotically modified microbiome complex plays the critical role in such preventive effects on Sharpey fibre^39,42,56–59^. The possible effect of such PDL alterations on the mechanical resistance also needs to be investigated fora better understanding of the PDL remodelling in dental patients with AN and other eating disorders. It is imperative to determine whether the effects of AN on the PDL observed in animal models are also present in clinical patients. Additionally, examining the impact of probiotic supplementation on the outcome of dental treatment in these patients is essential. Furthermore, future research could explore the potential synergistic effects of combining probiotics with other adjunctive treatments such as paraprobiotics, postbiotics, prebiotics, lysates, omega-3 fatty acids, vitamin C and D, ozone and light therapy. These approaches have previously demonstrated positive impacts on periodontal health and anti-inflammatory properties, offering promising avenues for enhancing periodontal vitality under ABA-like conditions^47,48,51,60–66^.

Evaluating periodontal ligament tissues presents certain limitations due to the methods employed in this research. Micro-CT evaluation provides insights into the hard tissue structures but is constrained in assessing the soft tissue aspects of the PDL. Meanwhile, histological analysis offers a more qualitative approach to examining PDL tissues; however, it is subject to variability influenced by examiner-dependent factors such as the position, size, and shape of the region of interest. These limitations underscore the necessity for molecular analyses that could elucidate underlying inflammatory responses or gene regulations contributing to observed phenomena. Such analyses are crucial for a comprehensive understanding and warrant further investigation in future studies. Additionally, while the ABA model serves as a useful proxy for studying lifestyle aspects associated with anorexia nervosa, it does not encompass the psychological dimensions of this condition. Consequently, this model may not fully replicate patient conditions, potentially leading to discrepancies between effects observed in animal models and those experienced by patients with anorexia nervosa.

This pioneering study of periodontium remodelling in the ABA rat model, simulating AN, revealed significant thinning and degradation of the PDL, while the morphology of the alveolar bone remained intact in ABA rats. Although probiotic treatment did not reverse the reduced dimensions of the PDL, it maintained the vitality and collagen structure of the PDL fibre bundles, comparable to the healthy group, irrespective of dietary conditions. These effects were consistent between the maxilla and mandible.

These findings highlight the need for further investigation into the structure of the PDL under AN and other dietary conditions, as well as the potential preventive effects of probiotics. Our findings highlight the importance of clinical awareness of this condition and the need for future studies aimed at understanding and refining therapeutic approaches in dental practice. Further research is required to elucidate the process of PDL remodelling under prolonged starvation, across different developmental stages and species, and in relation to mechanical stimuli in the periodontium.

## Data Availability

The data are available with the corresponding author and can be made available on reasonable request.
